# Surgical Management and Prognostic Prediction of Adenocarcinoma of Jejunum and Ileum

**DOI:** 10.1038/s41598-017-15633-w

**Published:** 2017-11-09

**Authors:** Xin Xie, Zhangjian Zhou, Yongchun Song, Chengxue Dang, Hao Zhang

**Affiliations:** Department of Surgical Oncology, The First Affiliated Hospital of Xi’an Jiaotong University, Xi’an, Shaanxi 710061 China

## Abstract

We conducted a retrospective study based on the Surveillance, Epidemiology, and End Results Program (SEER) database to establish a novel nomogram prognostic prediction system and to estimate the association between overall survival and prognostic factors, as well as to explore surgical treatment strategies for adenocarcinoma of the jejunum and ileum. A total of 883 patients from the SEER database were included in this study. Eight potential prognostic factors were included in a nomogram model and discriminatory power and accuracy were examined using the Harrell’s C-index and Akaike Information Criterion (AIC) index. In comparison with the AJCC TNM staging system, the nomogram prediction system was more accurate and homogeneous (Harrell’s C-index, 0.731 *vs*. 0.667; AIC index, 4852.9 *vs*. 4913.723). For surgical management, resection of more than 12 local lymph nodes could improve the likelihood of survival. This study demonstrates that our nomogram model is more accurate and homogeneous than the traditional AJCC TNM staging system, and proper surgical strategies for mesenteric lymphadenectomy improve overall survival.

## Introduction

Small bowel neoplasms, whether benign or malignant, are extremely rare^1^. The American Cancer Society estimates that there will be approximately 10190 new cases and 1390 deaths from small bowel neoplasms in 2017^2^. Although the mucosa surface of the small bowel accounts for more than 90% of the gastrointestinal tract, only 3% of neoplasms of the digestive tract originate in the small bowel^1^. Due to this relatively rare incidence, little clinical attention is paid to these neoplasms.

There are over 40 different histological types in both benign and malignant small bowel neoplasms^3^. Adenomas, lipomas and leiomyomas are the major types of benign neoplasms^4,5^, while adenocarcinomas and carcinoids (neuroendocrine tumours) are the most common types of the malignant tumours^6^. Several high-risk conditions also warrant additional attention due to their association with an increased risk of adenocarcinoma of the small bowel: familial adenomatous polyposis (FAP), Lynch syndrome, Peutz-Jeghers syndrome and Crohn’s disease^7–10^.

In comparison with other gastrointestinal neoplasms, small bowel tumours always present as non-specific complaints such as irregular abdominal pain, diarrhoea, gastrointestinal bleeding or intestinal obstruction, making it difficult to correctly diagnose the neoplasm^11^. Benign tumours often remain asymptomatic and are discovered either during surgery or occasionally during an autopsy. In recent years, the development of advanced imaging equipment and techniques enables the use of more reliable and accurate methods to detect these lesions in the small bowel^12^.

Nomogram, a graphical mathematical model that estimates several clinical factors in an integrative way, is considered to be a reliable clinical outcomes prediction tool^13,14^. After including significant risk factors, a nomogram algorithm can statistically estimate and predict several clinical endpoint events, such as prognosis and overall survival^15,16^. To date, nomogram models have been applied to prognostic prediction in many types of cancers, such as nasopharyngeal carcinoma (NPC), small cell lung cancer (SCLC), gynaecologic malignancies and gastrointestinal tumours, and are considered to be a more precise model than current staging systems^17–20^. Unfortunately, prognostic predicting tools for small bowels malignancies are rare.

Regarding small bowels malignancies, several studies have explored several prognostic factors, including race, gender, age, marital status, TNM staging and cancer-specific survival rate^21–24^. A number of studies have reported an association between the number of mesenteric lymph nodes (LNs) resected and patients’ prognosis^25,26^. Although lymphadenectomy is recommended in the National Comprehensive Care Network (NCCN) guidelines^27^, the impact on prognosis, particularly for patients with adenocarcinoma of the jejunum and ileum, is still undetermined. In our study, using the Surveillance, Epidemiology, and End Results (SEER) database, an open cancer statistical database established by the National Cancer Institute (NCI)^28^, we explored several prognostic factors and the surgical management of mesenteric lymph nodes of adenocarcinoma of the jejunum and ileum.

## Results

### Demographic and clinicopathological characteristics

Overall, 883 patients with adenocarcinoma of the jejunum and ileum were identified in the study cohort. Within this cohort, approximately half were male (n = 501, [56.7%]). The median age at diagnosis was 63 years (ranged from 20 to 100 years old), with average age of 62.88 ± 12.47 years. Regarding clinicopathologcal characteristics, half of the patients (n = 490, [55.5%]) were diagnosed with jejunum adenocarcinoma, while the remaining 393 patients were diagnosed with ileum adenocarcinoma. In 492 patients (55.7%), the adenocarcinoma had invaded the serosa, in the remaining 308 patients (34.9%), the tumour had invaded the serosa. Only 23.1% of patients (n = 204) received a lymph nodes examination. Additionally, 186 patients (21.1%) were diagnosed with distant metastasis, and nearly 283 patients (33%) were estimated to have poorly or undifferentiated adenocarcinoma of the jejunum or ileum.

### Overall survival and prognosis predictive factors for patients with adenocarcinoma of the jejunum and ileum

In this study, 10 clinicopathological factors were selected as potential prognosis predictors from the SEER database: age at diagnosis, gender, race, marital status, status of primary tumours (T stage), examined lymph nodes, lymph nodes status (N stage), histological grade, status of distance metastasis (M stage) and tumour locations (Table 1). Using a Cox proportional hazards regression model, seven factors were identified as associated with overall survival: age, marital status, T stage, N stage, number of lymph nodes examined, M stage and histological grade. Except for the histological grade, all factors were significantly associated with overall survival in multivariate analysis (*P* < 0.05).

**Table 1:** The univariate and multivariate analysis of adenocarcinoma of jejunum and ileum.

	N	Percentage (%)	Univariate		Multivariate	
			5-year Overall Survival	*P* value	*P* value	Hazard Ratio
Age						
Mean(SD)	62.88 ± 14.27					
Median(Range)	63 (20–100)					
Less than 50	162	18.3	58%			
50–75	540	61.2	49%			
More than 75	181	20.5	38%	<0.001	<0.001	1.232–1.695
Gender						
Male	501	56.7	48%			
Female	382	43.3	48%	0.752		
Race						
White	672	76.1	49%			
Black	163	18.5	45%			
Other	48	5.4	44%	0.388		
Marital status						
Single	360	40.8	42%			
Married	523	59.2	53%	0.001	0.017	0.806–0.979
T Stage						
T1	30	3.4	71%			
T2	53	6	75%			
T3	492	55.7	50%			
T4	308	34.9	39%	<0.001	0.002	1.009–1.043
Examined lymph nodes						
Less than 12	575	65.1	44%			
More than 12	308	34.9	55%	0.022	0.005	0.589–0.910
Lymph nodes status						
N0	522	59.1	56%			
N1	218	24.7	43%			
N2	143	16.2	28%	<0.001	<0.001	1.207–1.579
Grade						
G1	91	10.3	60%			
G2	509	57.6	49%			
G3	267	30.2	41%			
G4	16	1.8	0%	<0.001	0.057	0.996–1.354
Distance metastisis						
M0	697	78.9	58%			
M1	186	21.1	15%	<0.001	<0.001	1.103–1.152
Tumor location						
Jejunum	490	55.5	50%			
Ileum	393	44.5	46%	0.174		

### Nomogram prediction system for patients with adenocarcinoma of the jejunum and ileum

**Figure 1:**
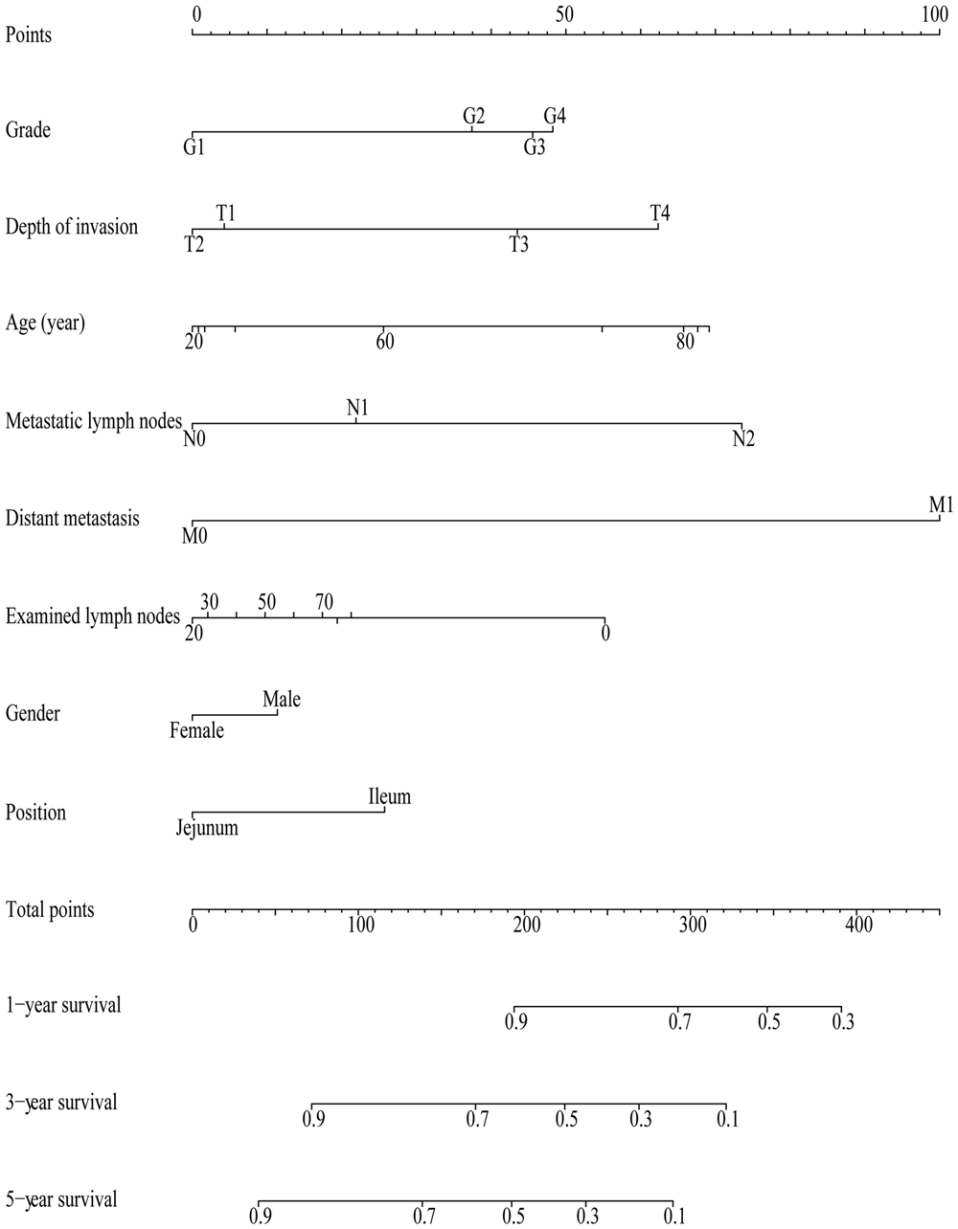
Nomogram predicted 1- to 5-year overall survival using eight available clinical characteristics.

**Figure 2:**
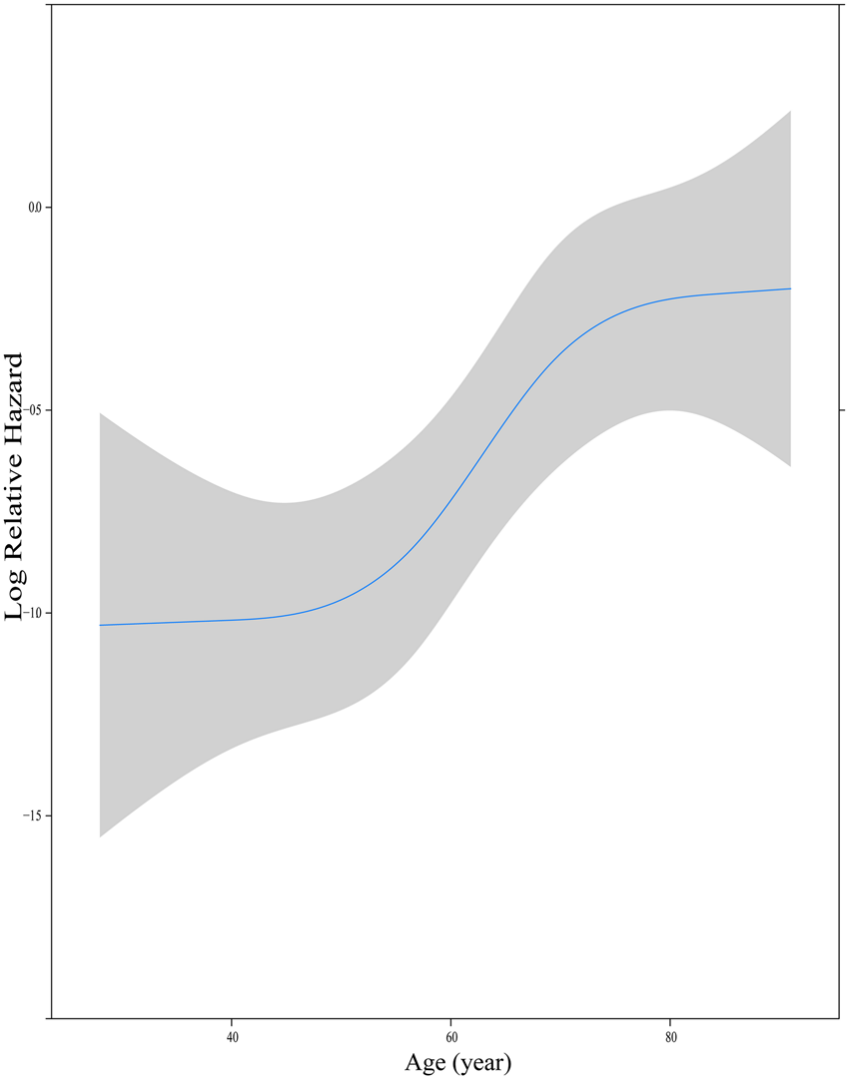
Transformation of continuous variables (Age) in univariate analysis using restricted cubic splines.

The nomogram prediction system was a novel model to estimate overall survival based on eight prognostic factors: histological grade, the TNM stage, age at diagnosis, number of lymph nodes examined, gender and primary tumour site. Each factor was ascribed a weighted point and the total points implied the prognosis. For example, 70 years of age was associated with 53 points, female was associated with zero points, depth of invasion (T_2_) was associated with zero points, no metastasized lymph nodes (N_0_) was associated with zero points, 30 examined lymph nodes was associated with three points, well-differentiated adenocarcinoma was associated with zero points, and distant metastasis was associated with 100 points, leading to a total score of 156. In addition, for each patient with adenocarcinoma of the jejunum or ileum, more total points in nomogram model indicated a worse prognosis. The factors and final nomogram model that estimate overall survival are shown in Fig. 1. Given that age was a non-linear prognostic factor, the restricted cubic spline (RCS) curve and hazard ratio are shown in Fig. 2.

**Figure 3:**
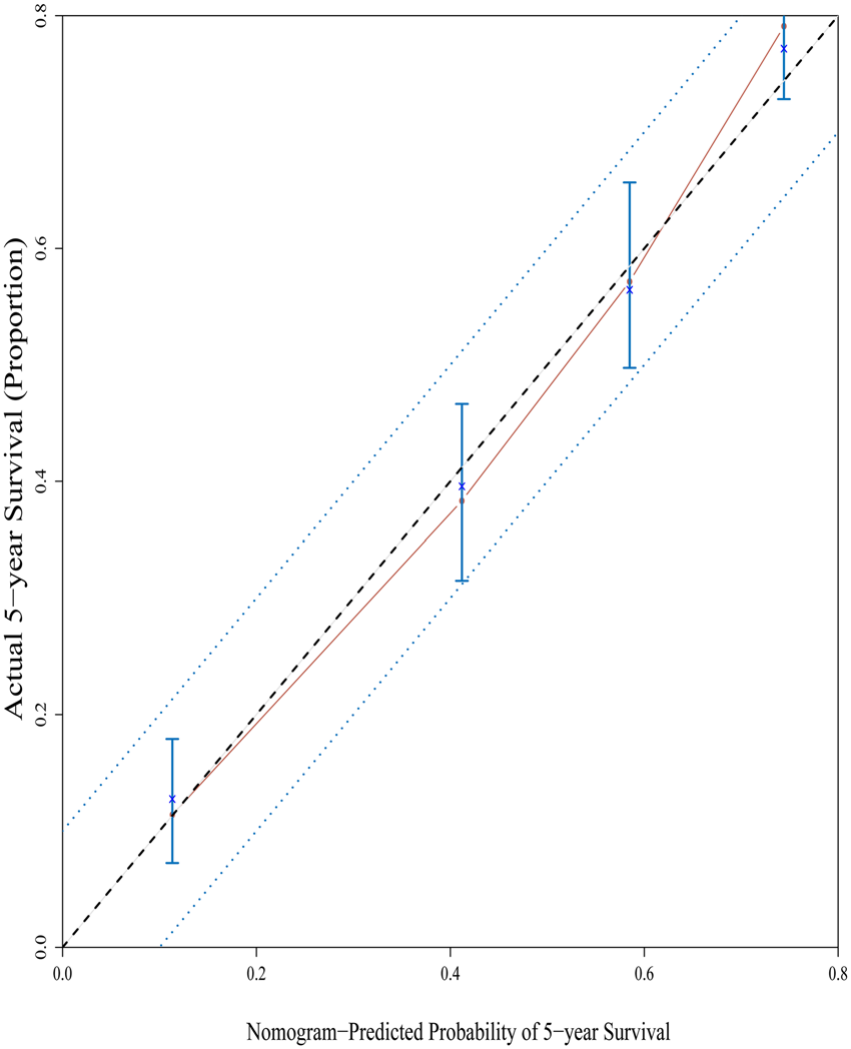
The calibration of the nomogram predicted system. Nomogram predicted probability of overall survival was plotted on the x-axis, actual overall survival was plotted on the y-axis. All predictions lie within the 10% margin of error (within the blue dots line).

To evaluate the predictive accuracy in both the nomogram prediction system and traditional AJCC TNM staging system, the Harrell’s C-index and AIC index were calculated. For the nomogram model, the Harrell’s C-index was 0.731 versus 0.667 in AJCC TNM system, indicating that our nomogram model was more accurate or discriminative in comparison with the traditional AJCC TNM staging system. Figure 3 presents the calibration plots of 5-year survival in the nomogram model, which revealed an overall survival predicted by nomogram model that closely approached the actual survival and was within a 10% margin of error. The AIC index was also calculated to avoid overfitting the prognostic models, and was 4852.9 for nomogram model and 4913.723 for AJCC TNM system, respectively. These results demonstrated that the nomogram model did not overestimate actual overall survival in this cohort and could be a better prognosis prediction system than the traditional AJCC TNM staging system.

### Surgical management of adenocarcinoma of the jejunum and ileum

**Figure 4:**
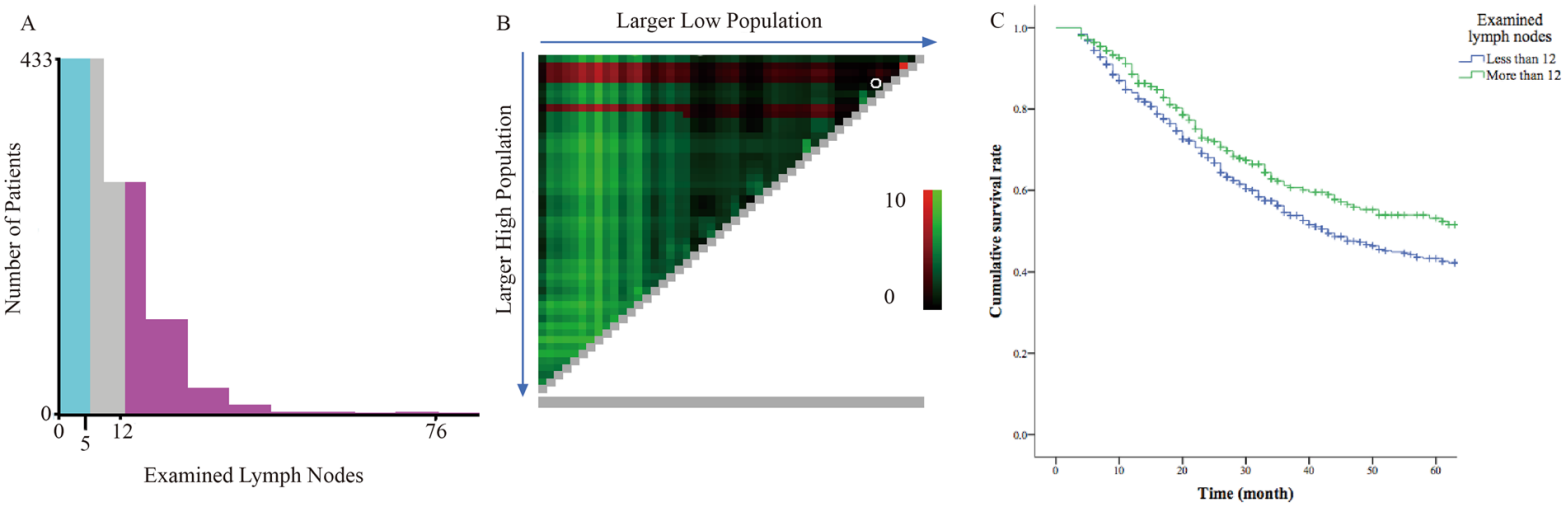
(**A**) The distribution of number of patients with adenocarcinomas of jejunum or ileum according to lymph nodes examined. (**B**) X-tile plots for number of lymph nodes constructed by patients with adenocarcinomas of jejunum or ileum. The plots show the χ2 log-rank values produced, dividing them into 2 groups by the cutoff point 12. (**C**) Kaplan-Meier survival curves of cumulative survival of patients with adenocarcinomas of jejunum or ileum.

Proper and beneficial surgical management strategies for patients with adenocarcinoma of the jejunum and ileum are still unclear. As mesenteric lymph nodes metastasis could be a vital factor in both prognostic prediction and therapeutic strategy, we explored the correlation between overall survival and the number of lymph nodes intraoperatively resected. In our study we analysed this correlation using an X-tile test. This test was used to determine the optimal cutoff point for predicting cancer-specific survival according to the number of lymph nodes examined. We observed that a resection of 5 or 12 lymph nodes could significantly improve overall survival (Fig. 4A and B). In consideration of the biological behaviour of adenocarcinoma of the jejunum and ileum and the guidelines for other gastrointestinal adenocarcinomas^29^, resection of more than 12 local lymph nodes could benefit patients with adenocarcinoma of the jejunum and ileum. In our study, these patients had improved cancer-specific survival (*P* < 0.05) (Fig. 4C).

## Discussion

Malignant neoplasms of the jejunum and ileum, particularly adenocarcinomas, are difficult to diagnosis and estimate before proper therapies because of their rare incidence and asymptomatic characteristics. Based on the selected risk factors, we assessed the prognosis of patients with adenocarcinoma of the jejunum and ileum using our newly established nomogram prediction model, which demonstrated higher accuracy and improved homogeneity as compared to traditional TNM stages. Notably, we determined that patients who underwent lymphadenectomy for more than 12 local lymph nodes could improve 5-year survival (Fig. 4C). This trend was in accordance with previous studies for patients with neuroendocrine tumours of the small bowel^26,30^.

In this study, we investigated several clinical characteristics that could potentially be prognostic predictors and survival outcomes of patients with adenocarcinoma of the jejunum and ileum. Consistent with previous studies, young age was a positive prognosis prediction factors in malignant tumours of the small bowel^22,31^. Additionally, marital status could be considered to be a protective factor, as we observed a Hazard Ratio of approximately 0.90 in married patients in comparison with single patients. However, in our study, we did not identify any significant differences in gender, race or locations of tumours, which might be other potential predictors in further investigations.

To analyse the association between survival outcomes and the potential prognostic factors, we utilized a nomogram prediction model as a novel prognosis prediction system and estimated the accuracy and homogeneity of both the nomogram model and the traditional AJCC TNM system. Based on the prognostic data of 883 patients who underwent surgery, we analysed eight factors to estimate the prognostic outcomes of those patients with adenocarcinoma of the jejunum and ileum. In the nomogram model, each factor was ascribed a weighted point to evaluate the effect on overall prognosis and the sum of the weighted points was considered to be an integrated factor to analyse the association between these prediction factors and overall prognosis: a higher scores indicated a worse prognosis. Furthermore, we examined the prediction accuracy and homogeneity of the nomogram model in comparison with the traditional AJCC TNM system. Harrell’s C indices and AIC indices were calculated, the results suggested our nomogram model was relatively close to the actual survival and more homogeneous (AIC index of 4852.9 for the nomogram model versus 4913.723 for the AJCC TNM staging system) than the traditional TNM system.

Surgical treatment is considered to be an important therapeutic strategy in gastrointestinal tumours^27^. Due to the rare incidence of adenocarcinoma of the jejunum or ileum, treatment and management strategies are still controversial. In previous studies, surgical excision has been demonstrated as a proper first-line therapy for patients with small bowel neoplasms, especially in carcinoid tumours of the small bowel and adenocarcinomas of the duodenum^32,33^. In surgery, local lymph nodes, including mesentery lymph nodes, are considered to be one of the essential factors tightly correlated with overall rates of survival. In our study drawing from a cohort of SEER database patients, we observed that lymphadenectomy of local lymph nodes could potentially predict prognosis. For patients with no nodal metastasis, intraoperativly increasing lymph nodes examination was significantly associated with an improvement in survival. Previous studies have shown that ≥ 15 lymph nodes resected for adenocarcinoma of the duodenum and ≥ 8 lymph nodes resected for carcinoid of the small bowel were positively correlated with overall survival^26,34^. In alignment with these studies, we analysed the clinicopathological data of 883 patients with adenocarcinoma of the jejunum and ileum and found that lymphadenectomy for more than 12 lymph nodes was significantly associated with positive patient prognosis (*P* < 0.022). This result also demonstrated that local lymph node estimation was essential for both stage assessment and the prognosis prediction system. Local lymph nodes identification is vital for both survival prediction and the management of therapies for adenocarcinoma of the small bowel. Because of its potentially metastatic behaviour, in adenocarcinoma of the duodenum, pancreaticoduodenectomy combined with extant lymphadenectomy, such as in patients with > 15 lymph nodes resected as mentioned previously, is essential and necessary for a better prognosis. In contrast, due to the rare incidence of adenocarcinoma of the jejunum and ileum invading contiguous organs, wide excision of tumours is easier to process. However, our study still holds the view that extant lymphadenectomy for more than 12 lymph nodes could improve prognosis for patients with adenocarcinoma of the jejunum and ileum.

Although increasing lymph nodes resection for patients with small bowel neoplasms might improve survival, challenges still exist during lymphadenectomies. Compared with other gastrointestinal tumours, adenocarcinoma of the jejunum or ileum presents more atypically and in emergency scenarios, such as with intestinal obstruction or bleeding, which makes it difficult for surgeons to identify the status of local lymph nodes. Additionally, because of stimulation from growth factors secreted from tumours or mesentery, fibrotic reaction could result leading to encasement of mesenteric vessels^26^. Excessive resection could damage these vessels and threaten the blood supply to the small bowel, leading to an incomplete local mesenteric lymphadenectomy of more than 12 lymph nodes.

It should be noted that multidisciplinary therapies, such as surgical resection combined with adjuvant chemotherapies, have been widely utilized for suitable patients with adenocarcinoma of the jejunum or ileum. Several clinical trials are exploring proper chemotherapeutic strategies and other factors impacting the efficacy of various chemotherapy reagents^35,36^. For instance, Gao, *et al*. elucidated that patients with Stage II colorectal cancer (CRC) could gain survival benefits from fluorouracil-based chemo reagents by utilizing a novel cancer hallmark-based gene signatures sets (CSS sets) model based on analysing 1005 patients with Stage II CRC from 13 cohorts^37^. Thus, precise and robust prognostic predictors are required and need to be further explored for multidisciplinary therapeutic management, particularly for adjuvant chemotherapy. For patients with Stage IV adenocarcinoma, peritoneal metastases (PM) occurs more easily in the jejunum or ileum than in duodenum because of its unique biological behaviour in its invasion into the peritoneum^38^. Systematic therapies for Stage IV adenocarcinoma of the jejunum or ileum warrant further investigation, whereas in case of local lesions of adenocarcinoma combined with PM, the proper therapeutic strategy is cytoreductive surgery followed by hyperthermic intraperitoneal chemotherapy (HIPEC), benefitting patients with PM or pseudomyxoma peritonei (PMP)^39^.

In addition, for prediction algorithms which are applied for patients with malignant tumours, accuracy and concordance or homogeneous are wildly considered. In comparison with the traditional TNM staging system, which only considers tumour size and extension (T stage), lymph nodes involvement (N stage) and distant metastasis (M stage)^17^, a nomogram prediction model with multiple factors has major benefits. Except for clinicopathological predictors, algorithms focusing on metastasis-driving genes or mutant gene signal pathways could also be precise prognosis prediction models based on the statistical analysis of tumour gene microarrays and Multiple Survival Screening (MSS) algorithm^40^. In breast cancer, the *PIK3CA*-mutated signalling pathway and 26 S proteasome genes were proved to be associated with patients’ prognosis by gene microarrays and signalling network analysis^41,42^. Therefore, a potential prognostic prediction model combining clinicopathological characteristics and bioinformatics approaches, such as genome sequencing data and network analysis, could be a more accurate and robust model, which requires further exploration.

Although our study is based on 10 years SEER database data, there are still several limitations. First, this study utilizes retrospective population-based data across several specific states in the United States but not worldwide. Second, certain clinical details such as specific details of chemotherapy, the approaches of surgery or information on disease-free survival were not complete, which may influence the prognosis of patients in our cohort. Furthermore, the histological grade might cause bias due to relatively subjective diagnostic standards among different pathologists or clinicians.

In summary, our study demonstrated that the nomogram prognosis prediction model could estimate overall survival more accurately and with improved homogeneity as compared to the traditional AJCC TNM staging system. Additionally, our analysis elucidated that mesenteric lymphadenectomy for patients with adenocarcinoma of the jejunum or ileum could improve overall survival.

## Patients and Methods

### Patients

In our study, data collected included the demographic and clinicopathological characteristics and survival of patients with adenocarcinoma of the ileum and jejunum. All patients were identified between 2003 and 2014 in the SEER database. The inclusion criteria were follows: 1. Patients clinicopathologically diagnosed with adenocarcinoma of the ileum or jejunum; 2. Patients who underwent surgery and for whom exact pathological details were available; 3. Patients who survived for more than three months postoperatively. In our study, a signed SEER research data agreement form was provided to the SEER program and we were given approval to access and analyse SEER data.

### Statistical analysis

All patients were regrouped according to the 7^th^ AJCC TNM staging system^43^. Continuous data were presented as means ± standard deviations. Categorical variables were compared using the χ^2^ test or Fisher’s exact test. Continuous variables were compared using the Student’s t-test. Both univariate and multivariate Cox proportional hazard regression models were used to explore the association between all factors and overall survival. Overall survival was estimated using the Kaplan-Meier estimator, and differences in survival were examined using the log-rank test. Restricted cubic splines were applied to transform continuous non-liner prediction factors to fit the test statistic. X-tile analysis was used to determine the optimal cutoff point for predicting survival according to the number of lymph nodes examined in patients with adenocarcinoma of the ileum and jejunum. Preselected multiple potential factors were tested as nomogram parameters irrespective of significance. To estimate the accuracy and identification abilities of those predictors, Akaike’s Information Criterion (AIC) and Harrell’s C statistic were used in this study. All statistical tests were two-sided, and *P* values < 0.05 were considered to be statistically significant. Statistical analyses were performed using SPSS 13.0 and R software version 3.3.0 (http://www.r-project.org) with the “SEERaBomb”, “rms” and “AICcmodavg” packages.
